# Supplementary material for: Nitrogen Use Efficiency in an Agrisilviculture System with Gliricidia sepium in the Cerrado Region
Source: Plants (Basel). 2023 Apr 14;12(8):1647. doi: 10.3390/plants12081647 (PMC10143777; doi:10.3390/plants12081647)
Supplement: Supplementary file 1 [file plants-12-01647-s001.zip › plants-2277522-supplementary.pdf]

Supplemental material

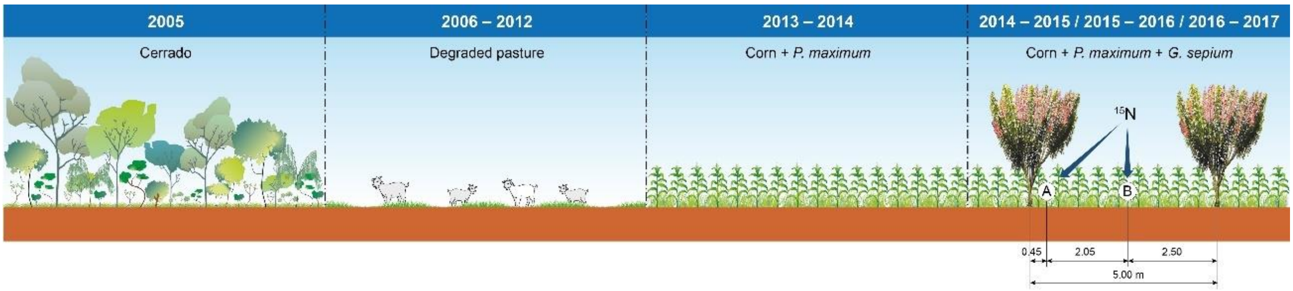

Figure S1. Schematic representation of the experimental area along the years highlighting the isotopic study in the 2016/2017 cropping season.

Table S1. Soil chemical and physical properties before establishing the agrisilvicultural system at the 0.00 – 0.20 m layer.

N	SOC	P	pH	K^+	Ca^{2+}	Mg^{2+}	H + Al	SB	V	m	Sand	Silt	Clay	Bd
g kg^{-1}	g kg^{-1}	mg dm^{-3}	(H_2O)			cmol$_c$ kg^{-1}			%	%		g kg^{-1}		Mg m^{-3}
1.2	18.2	1.4	5.2	0.1	2.6	1.0	4.8	3.6	42.7	2.5	45	360	595	0.82

N: total nitrogen (Kjeldahl method); SOC: soil organic carbon (Walkley-Black method); P e K: extracted with Mehlich-1 (HCl 0.05 mol L^{-1}+ H_2SO_4 0.0125 mol L^{-1}); Ca e Mg: extracted with 1 mol L^{-1} KCl; H + Al: extracted with calcium acetate buffer solution at pH 7.0; SB = sum of bases; V: base saturation; m: aluminum saturation; Bd: soil bulk density

Table S2. Description of the seasonal agricultural practices during the experiment study period.

**Season 2012/2013**
- Soil was plowed and harrowed with 1.5 t ha^{-1} of dolomite lime (100% acid neutralizing potential - ANP).<br>- 87 kg ha^{-1} P_2O_5 ("corrective fertilization") as single super phosphate.<br>- Corn (hybrid AG 1051) planted on January 03, 2013.<br>- *P. maximu*m cv. Massai planted on January 04, 2013.<br>- Fertilization at planting: 20 kg N ha^{-1} +100 kg P_2O_5 ha^{-1} + 84 kg K_2O ha^{-1}.<br>- Sidedressing fertilization: 50 kg N ha^{-1} +40 kg K_2O ha^{-1} on February 2013.<br>- Corn harvest: July 2013.<br>- To simulate the grazing in the integrated system, at the end of July, 2013, after the corn harvest, animals (sheep; stocking rate of 10 AU/ha) enter the experimental area to graze on *P. maximum* over 20 days.
**Season 2013/2014**
- Corn (hybrid LG 6030) planted on December 26, 2013.<br>- Fertilization at planting: 30 kg N ha^{-1}+120 kg ha^{-1} P_2O_5+ 64 kg ha^{-1} K_2O.<br>- Sidedressing fertilization: 65 kg N ha^{-1} (January 2014) and 65 kg N ha^{-1} + 65 kg K_2O ha^{-1} (February 2014).<br>- Insecticide Lufenuron (100 g ha^{-1}) for *Spodoptera frugiperda.*<br>- Corn harvest: July 2014.
**Season 2014/2015**
- Application of 0.5 t ha^{-1} of dolomite lime (100% ANP).<br>- Corn (hybrid LG 6030) planted on December 18, 2014.<br>- Fertilization at planting: 20 kg N ha^{-1} + 150 kg P_2O_5 ha^{-1} + 80 kg K_2O ha^{-1}.<br>- Sidedressing fertilization: 80 kg N ha^{-1} (January 2015) and 60 kg N ha^{-1} + 60 kg K_2O ha^{-1} (February 2015).<br>- *Gliricidia sepium* was planted in January 2015 with 50 g simple superphosphate + 25 g of KCl and 10 g of fritted trace elements (FTE BR12).<br>- Corn harvest: June 2015.
**Season 2015/2016**
- Fertilizer applied to the vertical projection ground of the *Gliricidia sepium* crown – 50 g simple superphosphate + 25 g of KCl and 10 g of fritted trace elements (FTE BR12).<br>- Application of herbicide Paraquate (200 g L^{-1}) (1.5 L ha^{-1}) on December 2015.

- Corn (hybrid LG 6030) planted on December 17, 2015.
- Fertilization at planting: 30 kg N ha^{-1} + 150 kg ha^{-1} P_2O_5 + 50 kg ha^{-1} K_2O + 66 kg ha^{-1} of FTE BR12.
- Sidressing fertilization: 90 kg N ha^{-1} + 30 K_2O ha^{-1} (January 2016) and 60 kg N ha^{-1} + 60 kg K_2O ha^{-1} February 2016).
- Corn harvest: May 2016.

Safra 2016/2017

- *Gliricidia sepium* pruning to a height of 1.0 m from the soil in December 2016. The biomass (a mix of stem and leaf) that resulted from the pruning was distributed in the plots: 1,196 kg ha^{-1}, 1,600 kg ha^{-1} and 1,804 kg ha^{-1} in plant densities 667, 1000 e 1333 plants of gliricidia ha^{-1}, respectively.
- *P. maximum* was cut in December 2016 before the corn planting.
- Application of herbicide Glysphosate (480 g L^{-1}) subdose on the forage (2 L ha^{-1}) on January 2017.
- Establishment of the microplots for the application of the ^{15}N-enriched ammonium sulfate.
- Corn (hybrid LG 6030) planted on January 17, 2017.
- Fertilization at planting: 350 kg ha^{-1} of blended NPK 4-30-16 (14 kg N ha^{-1}, 105 kg P_2O_5 ha^{-1}, 56 kg K_2O ha^{-1}).
-Sidedressing fertilization: 104 kg N ha^{-1} and 60 kg K_2O ha^{-1} (February 2017).
- Corn harvest: July 2017.
- *P. maximum* harvest and yield measure: July 2017.
